# Temperate Forest Floor Bryophytes Functionally Respond to Small‐Scale Variability in Water, Light and Nutrient Availability

**DOI:** 10.1002/ece3.71839

**Published:** 2025-07-31

**Authors:** T. J. Deilmann, M. Bernhardt‐Römermann, J. Hentschel, P. Gros, C. Römermann

**Affiliations:** ^1^ Institute of Biodiversity, Ecology and Evolution Friedrich Schiller University Jena Jena Germany; ^2^ Senckenberg Institute for Plant Form and Function Jena Jena Germany; ^3^ German Centre for Integrative Biodiversity Research (iDiv), Halle‐Jena‐Leipzig Leipzig Germany; ^4^ Thüringer Landesamt für Landwirtschaft und Ländlichen Raum (TLLLR) Jena Germany

**Keywords:** environmental variability, functional traits, growth form, moss, small‐scale response, water balance

## Abstract

Bryophytes form an integral component in numerous ecosystems. They impact ecosystem processes by regulating water, carbon, and nutrient input into the soil, making them an ecologically significant but understudied group of plants. To understand ecosystem processes, functional traits offer a suitable tool as they reflect plant performance and strategies that respond to changes in the environment. Functional traits, however, have been hardly studied and are still poorly understood in bryophytes, limiting the understanding of functional responses to environmental variability and future change. Therefore, we here measured 10 functional traits related to water balance (e.g., leaves per cm, branching density, water uptake capacity) and productivity (e.g., shoot length, in situ fluorescence, specific shoot area) and related them to environmental variability for eight common forest floor bryophyte species in two temperate coniferous forests. We tested how well these traits respond to small‐scale variability in water, light, and nutrient availability. Multivariate analyses showed a large variation in trait composition of the investigated species, mainly driven by growth form (pleurocarpous vs. acrocarpous), while the impact of forest type (Norway spruce vs. Scots pine) on trait composition seemed less important. Mixed effects models across all species revealed that traits were very sensitive to within‐forest small‐scale variability; for example, leaves per cm or in situ fluorescence were positively related to increasing plot‐level characteristics such as leaf area index and throughfall, again with growth form‐specific responses. We further found intraspecific trait variation for the most dominant bryophyte species, indicating considerable phenotypic plasticity. We conclude that moss trait variability is more linked to growth form than to forest type, and that both bryophyte communities and individual species are functionally sensitive to small‐scale environmental variability. We therefore emphasize including bryophytes and growth form as a functional group more specifically in functional response studies.

## Graphical abstract

Bryophytes are an integral component of many ecosystems, but their functional ecology is little studied and therefore poorly understood. We related measurements of 10 functional traits for over 400 samples from common forest floor bryophytes to small‐scale environmental variability and found that bryophyte traits respond highly sensitively. We encourage including them in future functional studies to improve the understanding of ecosystem functioning and change.

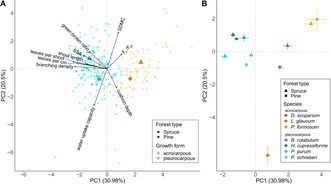

## 1 Introduction

Bryophytes are an integral component of numerous ecosystem functions and services (Bates 2008; Cornelissen et al. 2007; Eldridge et al. 2023; Glime 2024; Oechel and Van Cleve 1986; Turetsky et al. 2012). They regulate water input through interception and influencing water flow paths (Gall et al. 2024; Porada et al. 2018), influence carbon cycling through contributing to net primary productivity and acting as carbon sinks (Eldridge et al. 2023; DeLucia et al. 2003; Turetsky 2003), and impact nutrient cycling through biological N_2_ fixation and nutrient uptake and conservation (Cornelissen et al. 2007; DeLuca et al. 2002; Hupperts et al. 2021; Liu et al. 2020). As they form dense communities on the ground, they function as an essential linking layer between surface and subsurface, thereby strongly influencing whole ecosystems. Since bryophytes grow along small‐scale microclimatic gradients (Proctor et al. 2007), studying responses at this scale will be the first step to find patterns and mechanisms before scaling up to larger scales. To understand how bryophytes respond to ongoing climate change and predicted increasingly frequent disturbances (IPCC 2023), functional trait approaches are promising because they reflect plant‐ and eventually ecosystem performance (Violle et al. 2007). Therefore, we here measured 10 functional traits related to water balance and productivity for eight common forest floor bryophyte species over small‐scale environmental variability in two coniferous forests in Central Germany.

Many studies have focused on the response of bryophytes by evaluating changes in presence/absence data of species or species composition (Becker Scarpitta et al. 2017; Økland et al. 2023; Riffo‐Donoso et al. 2021). Less attention has been paid to the functional changes so far (Deilmann, Christiansen, et al. 2024; St. Martin and Mallik 2017; Van Zuijlen et al. 2023). However, studies on functional traits give crucial information about ecosystem processes and functioning which need to be understood in detail to assess current states and potential future changes of ecosystems. There are some comprehensive trait databases available but they mostly cover categorical or life history traits (e.g., Bernhardt‐Römermann et al. 2018; Van Zuijlen et al. 2023) making it difficult to use them for studies on gradual changes in the environment.

Only a few studies have related functional responses to environmental variation at small scales so far, although trait relationships have been shown to vary at the microhabitat scale: with higher microhabitat irradiance, mosses showed smaller leaves with smaller cells and thicker cell walls, and higher assimilation and respiration rates per area (Waite and Sack 2010). Even within a single hummock, *Sphagnum* L. showed large variation in water retention, capitulum mass, and total biomass (Oke and Turetsky 2020). As more emphasis has been placed on physiological variables such as nutrient and chlorophyll content, assimilation, respiration, or light response curves (Grau‐Andrés et al. 2022; Marschall and Proctor 2004; Wang et al. 2014), there is a lack of morphological traits that are related to essential eco‐physiological functions for bryophytes such as water balance or overall leaf surface area.

As poikilohydric organisms without roots, bryophytes take up most of their nutrients and water through their leaf surfaces by direct absorption (Bates 2008; Glime 2022). The most important environmental drivers for bryophyte productivity are therefore linked to water, light, and nutrient availability, which are undoubtedly strongly interdependent: increasing water availability and temperature generally increase microbial activity and hence nutrient availability (Muscolo et al. 2014). Increasing light intensity and temperature decrease soil moisture but increase evapotranspiration, making water the limiting resource. This results in a trade‐off between responses to water and light availability, especially for sensitive poikilohydric organisms. For instance, on the one hand, ground‐dwelling forest bryophytes are shade tolerant due to their low photosynthetic capacity, making them, however, more susceptible to photoinhibition on the other hand (Marschall and Proctor 2004; Proctor and Bates 2018). The high desiccation tolerance, one of the most intriguing features of bryophytes, leads to survival in extreme light or warm conditions with very limited water, but results in long time periods of inactive metabolism, hence being unproductive (Proctor et al. 2007).

These trade‐offs lead to the emergence of many different plant strategies. Some adaptations in morphological traits, for example, led to the ability to store more external capillary water in, for example, sheathing leaves, paraphyllia, or between shoots. By this, certain species possessing these traits have an increased water balance and productivity by increased time the cells are turgid and thus able to photosynthesize and grow (Proctor 2008; Schofield 1981). Similarly, moderately shaded, moist environments promote the growth of frequently branched wefts as a resource acquisition strategy, such as capturing nutrients from throughfall (Bates 1998).

It has been shown that different growth forms respond differently to variations in the environment. For instance, mostly prostrate and frequently branched pleurocarpous mosses mainly occur in shady and humid habitats, while acrocarpous mosses, that mostly grow erect and form dense cushions or turfs, tolerate more sun‐exposed and xeric habitats (Bates 1998), suggesting a higher water‐use efficiency. These different strategies are visible in morphological trait variation in growth forms (Bates 1998; Schofield 1981), making responses to environmental variability likely to differ between pleuro‐ and acrocarpous mosses.

To provide a better understanding of how functional traits respond to environmental variability, we selected 10 traits that are related to productivity and water balance: leaves per cm, leaves per shoot, shoot length, branching density, green:brown ratio, cushion depth, shoot dry matter content, specific shoot area, water uptake capacity, and in situ fluorescence. We measured those traits for 427 samples from eight forest floor bryophyte species on 30 plots covering small‐scale environmental variability within a spruce and a pine forest in Central Germany. Using this data set, we test the following hypotheses:

- Trait composition will differ between growth forms and forest types since the former reflects life strategies and the latter different environmental conditions;
- Trait values related to improved water balance (e.g., water uptake capacity), and microstructures for storing extra capillary water (by e.g., a higher number of leaves and branches), will decrease with increasing throughfall as there is less need to retain water;
- Trait values related to photosynthetic efficiency (e.g., *F*_v_/*F*_m_), and overall leaf surface (e.g., branching density, leaves per shoot, shoot length), will decline with increasing light availability to avoid photoinhibition as forest floor bryophytes favor shaded conditions;
- An increase in nutrient availability will generally promote growth and hence lead to a higher number of leaves, longer shoots, higher specific shoot area, and larger green:brown shoot ratio.

Answering these hypotheses will help understand small‐scale functional responses of bryophytes to the environment, and hence the role of bryophytes within temperate forest ecosystems.

## 2 Material and Methods

### 2.1 Study Site and Species Selection

**FIGURE 1:**
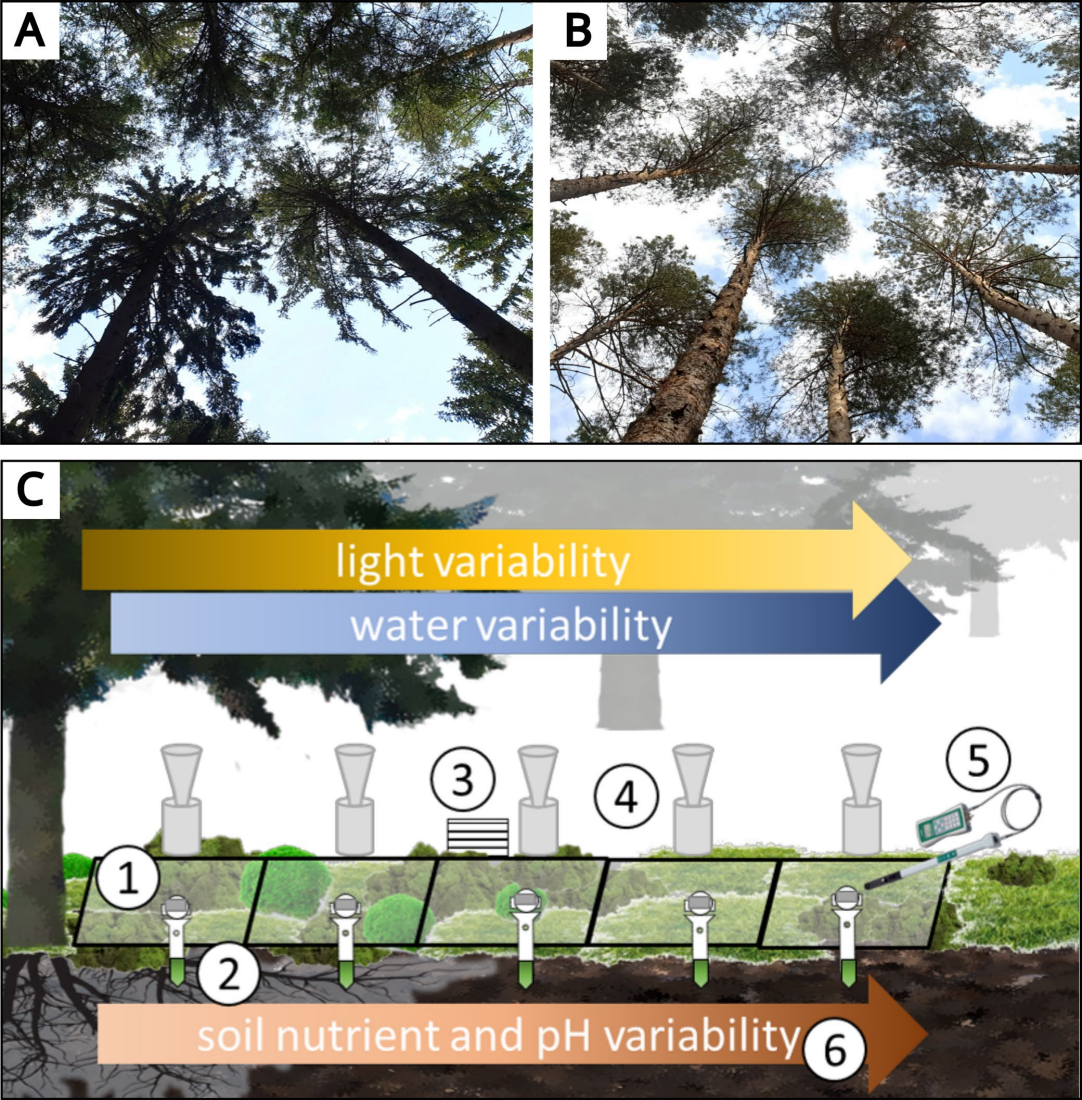
View from bottom to top of the heterogeneous canopy cover within (A) a spruce and (B) a pine forest. (C) Experimental design: Environmental variability on the forest floor was captured by recording establishing six transects in two forest types, consisting of five adjacent plots each (*N*_plots_ = 30), ranging from close to the stem to under a canopy gap. (1) Light intensity and temperature logger on top of (2) soil moisture and soil temperature logger, (3) air humidity logger, (4) precipitation throughfall collector, and (5) Leaf Area Index device. (6) Soil nutrients (C, N, P, K, Ca, Mg, Mn) and pH were measured once per plot. For more details on recorded variables, see Table 3.

To capture environmental variability, we established six transects of five adjacent plots of 60 cm × 60 cm each in two temperate coniferous forest types of the Saale‐Elster‐Sandsteinplatte Observatory in Thuringia, Central Germany, namely a Norway spruce ( *Picea abies* (L.) H.Karst.) and a Scots pine ( *Pinus sylvestris* L.) forest (*N*_plots_ = 30; Figure 1). Every transect ranged from close to the stem, that is, assumed low light transmission or high canopy cover, to a canopy gap, that is, assumed high light transmission or low canopy cover, spanning *c*. 3.5 m. All transects per forest type together constituted the captured environmental variability.

For the functional trait measurements, we selected five individuals per plot from all co‐occurring forest floor bryophytes within our transects that were abundant enough to be measured, resulting in 427 samples from eight common moss species in May 2022 (Table 1; henceforth, we use the terms “bryophyte” and “moss” interchangeably). We defined individuals as samples collected as far apart within a plot as possible. Sampling procedure included carefully extracting patches of whole gametophytes with both green and brown parts, storing them in a paper bag and air drying them. We are aware of differences in sample sizes between species which, however, represent natural occurrences reflecting long‐term processes and environmental variability. For storage and preservation until further measurements, we used the method for botanical collections of the Herbarium Haussknecht Jena, freezing them twice at −25°C for 2 weeks each, with 1 week at room temperature in between, which empirically leads to a better preservation than just air drying. Furthermore, we defined growth form as equal to perichaetial position, namely pleurocarpous and acrocarpous (La Farge‐England 1996). All pleurocarpous mosses in this study grow prostrate and all acrocarpous mosses grow erect. Species taxonomy follows (Euro+Med 2006–ongoing).

**TABLE 1:** Overview of the bryophyte species for which functional traits were measured, together with their growth form, occurrence on plots, and number of samples. Shaded species were additionally selected for species‐specific analyses based on their occurrence on most plots.

Species name	Family	Growth form	Presence in number of plots (spruce + pine)	Samples (spruce + pine)
*Brachythecium rutabulum* (Hedw.) Schimp.	Brachytheciaceae	Pleurocarpous	8 (8 + 0)	31 (31 + 0)
*Dicranum scoparium* Hedw.	Dicranaceae	Acrocarpous	30 (15 + 15)	118 (52 + 66)
*Hypnum cupressiforme* Hedw.	Hypnaceae	Pleurocarpous	26 (15 + 11)	87 (75 + 12)
*Leucobryum glaucum* (Hedw.) Ångstr.	Leucobryaceae	Acrocarpous	4 (2 + 2)	5 (3 + 2)
*Mnium hornum* Hedw.	Mniaceae	Acrocarpous	2 (2 + 0)	5 (5 + 0)
*Pleurozium schreberi* (Willd. ex Brid.) Mitt.	Hylocomiaceae	Pleurocarpous	24 (9 + 15)	90 (17 + 73)
*Polytrichum formosum* Hedw.	Polytrichaceae	Acrocarpous	18 (12 + 6)	56 (43 + 13)
*Pseudoscleropodium purum* (Hedw.) M. Fleisch.	Brachytheciaceae	Pleurocarpous	9 (9 + 0)	35 (35 + 0)

### 2.2 Functional Trait Measurements

We selected 10 morphological and physiological functional traits which we assumed to be related to productivity and growth, competition, and hydrologic balance. Table 2 summarizes the traits with their hypothesized functions and response to water‐, light‐, and nutrient availability.

**TABLE 2:** Summary of the recorded functional traits of ground dwelling forest bryophytes with their associated ecological functions, together with the assumed and measured responses to the water, light, and nutrient availability (avail.). Arrows indicate a positive (↑), negative (↓), unclear (↑↓) or no relationship (—). Unclear relationships can result from contrasting responses of environmental variables within or between forest types or growth forms. Colours represent whether hypotheses were supported (blue), not supported (red), or received ambiguous support (yellow). Dry weight in the definition refers to the air‐dried mass while fresh weight means the water saturated mass after immersion in distilled water as described in Section 2.

Functional trait	Definition	Unit	Ecological function	Assumed response to ↑ in			Measured response to ↑ in		
				Water avail.	Light avail.	Nutrient avail.	Water avail.	Light avail.	Nutrient avail.
Branching density	All counted branches of the green shoot, divided by its length	cm^−1^	Hydrologic balance, productivity	↓	↓	↑	—	↑	↑↓
Cushion depth	Maximum in situ measured depth of the bryophyte cushion, mat, or turf, depending on the species	cm	Hydrologic balance, micro‐climate	↓	↓	↑	↑	↓	↑
Green:brown ratio	Length of the green (photosynthetically active), divided by the brown (senesced) shoot part	—	Productivity, hydrological balance, decomposition	↑	↓	↑	↓	—	↓
Leaves per cm	Counted number of stem leaves per cm in an average part of the green shoot	cm^−1^	Hydrologic balance, productivity	↓	↓	↑	↑↓	↓	↑
Leaves per shoot	Leaves per cm times the length of the green shoot	—	Hydrologic balance, productivity	↓	↓	↑	↑↓	↓	↑
Shoot length	Maximum length of a complete stretched green shoot	mm	Productivity, competition	↑	↓	↑	—	↓	↑
In situ fluorescence (*F*_v_/*F*_m_)	Measured maximum quantum yield after 30 min dark adaptation	—	Productivity	↑	↓	↑	↑	↓	↓
Shoot dry matter content (SDMC)	Quotient of green shoot dry weight to its fresh weight	mg g^−1^	Investment in structural biomass, decomposition	↓	↓	↓	↑	↑	↑↓
Specific shoot area (SSA)	Quotient of green shoot area to its dry weight	mm^2^ mg^−1^	Productivity, competition	↑	↓	↑	—	↓	↑↓
Water uptake capacity (WUC)	Maximum WUC calculated as (Fresh weight − Dry weight)/Dry weight	g g^−1^	Hydrologic balance, micro‐climate	↓	↑	↑	—	↓	↑↓

“Shoot length” was measured by determining the length of the stretched green part of a shoot with a millimetre scale under a stereo microscope. Both the length of the photosynthetically active green and of the dead brown part of a shoot were recorded to calculate the “green:brown ratio”. To obtain the “branching density”, we counted all branches of the green part and divided by its length. Furthermore, we calculated “leaves per cm” by counting all stem leaves in a representative part of the green shoot over a standardised length of 5 mm and doubling the value. We did not count 1 cm directly in order to include species with small green shoot parts such as *Leucobryum glaucum* . Additionally, we calculated “leaves per shoot” by multiplying leaves per cm with (green) shoot length. While the former should reflect potential extra‐capillary water storage, “leaves per shoot” should rather reflect potential photosynthetic ability. “Cushion depth” was recorded in situ using a measuring stick with 5 mm resolution which was gently poked down through the cushion to the substrate. “Specific shoot area” (SSA) was calculated by dividing the area of five green shoots by their dry weight. The area was calculated using an image detection algorithm (M. Körschens, unpublished). We obtained “shoot dry matter content” (SDMC) by dividing the dry weight of five green shoots by their water saturated fresh weight after being soaked in distilled water for 5 min and carefully blotted with a paper towel, giving an approximate estimate of water content at full turgor (Proctor 2008). The “water uptake capacity” (WUC) was retrieved by adapting the formula from Michel et al. (2013): WUC = (fw − dw)/(dw) where dw was the dry weight of five representative green shoot parts while fw was their water saturated fresh weight. While WUC and SDMC are mathematically dependent, they represent different ecological functions, namely hydrological balance, and investment in structural biomass, respectively, which is why we included both. “In situ fluorescence” or *F*_v_/*F*_m_, a common measure of maximum photosynthesis efficiency of PSII, was measured using the chlorophyll fluorimeter Pocket PEA (Hansatech Instruments Ltd., UK) after at least 30 min of dark adaptation of the green shoot. To capture several states of metabolic activity and obtain a robust value, *F*_v_/*F*_m_ should be measured at different metabolic states. In our case, we averaged the values of two measurements: once in May when it was dry (precipitation sum ± standard deviation: 19.64 ± 0.06; DWD 2025) and we expected low photosynthetic activity, and once in early December when it was moist (52.73 ± 0.08) and we expected peak photosynthesis.

### 2.3 Environmental Characterisation

Table 3 gives an overview of all measured environmental variables with respective value ranges. In more detail, we characterized small‐scale environmental variability for each plot by biweekly measurements of precipitation throughfall volume with funnel type samplers, and leaf area index (LAI) of the aboveground canopy, that is, herb‐, shrub‐, and tree canopy, with the LAI 2200 (LI‐COR Inc., USA). Furthermore, continuous volumetric soil moisture content and soil temperature at 6 cm depth were recorded using TMS‐4 Standard loggers (TOMST s.r.o., Czech Republic), as well as temperature and light intensity on top of the TMS‐4 loggers with HOBO MX2202 loggers (Onset Computer Corporation, USA). Relative air humidity and dew point were continuously recorded close to the ground per forest type with HOBO MX2301A loggers (*N*_loggers_ = 3 per forest type; Onset Computer Corporation).

**TABLE 3:** Summary of the plot‐wise measured environmental variables and their value range representing the variability within each forest type (*N* = 15 each). For more details on methodology, see Section 2.

Variable		Unit	Device/method	Spruce forest, mean [min; max]	Pine forest, mean [min; max]	Links to
Aboveground	Dew point	°C	Onset HOBO MX2301A	8.13 [8.13; 8.13]^a^	8.84 [8.84; 8.84]^a^	Water
	Leaf Area Index	—	LI‐COR LAI 2200	2.65 [2.42; 2.93]	2.38 [1.6; 3.69]	Light
	Light intensity	lux	Onset HOBO MX2202	1563 [1399; 1681]	2314 [1700; 2775]	Light
	Precipitation throughfall	mm	Pluviometer	165 [128; 224]	170 [131; 196]	Water
	Relative air humidity	%	Onset HOBO MX2301A	79.3 [79.3; 79.3]^a^	80.6 [80.6; 80.6]^a^	Water
	Temperature	°C	Onset HOBO MX2202	12.1 [12.0; 12.3]	12.5 [12.3; 12.7]	Water/nutrient
Belowground	*Soil nutrients*					
	Total C	%	Combustion (DIN ISO 13878:1998‐11, VDLUFA I, A 4.1.3.1, 2016; Vario Max Cube, Elementar, Germany)	C: 34.4 [23.1; 46.1]	C: 26.8 [20.4; 35.6]	Nutrient
	Total N			N: 1.1 [0.67; 1.43]	N: 0.83 [0.65; 1.06]	
	Plant available P	mg kg^−1^	CAL extraction (VDLUFA I, A 6.2.1.1, 2012); photometry	73.7 [38; 116]	75.4 [50; 114]	Nutrient
	Plant available K	mg kg^−1^	CAL extraction; F‐AAS	419 [239; 632]	400 [307; 532]	Nutrient
	Plant available Mg	mg kg^−1^	Calcium chloride extraction (VDLUFA I, A 6.2.4.1)	195 [137; 276]	213 [166; 276]	Nutrient
	Plant available Ca	mg kg^−1^	Ammonia‐acetate extraction (VDLUFA I, A 6.2.4.1); ICP‐OES	1912 [856; 4059]	1926 [1562; 2580]	Nutrient
	Plant available Mn	mg kg^−1^	CAT extraction (VDLUFA I, A 6.4.1); ICP‐OES	326 [59.4; 499]	198 [94.3; 374]	Nutrient
	*Soil pH (H*_*2*_ *O)*	—	DIN EN 15933:2012‐11	4.03 [3.7; 4.4]	4.17 [3.7; 4.4]	Nutrient
	*Soil temperature*	°C	TOMST TMS‐4 Standard	11 [10.9; 11.2]	12.1 [11.6; 12.3]	Water/nutrient
	*Volumetric soil moisture*	vol%	TOMST TMS‐4 Standard	10.8 [4.19; 19.8]	19.4 [4.75; 24.5]	Water

Abbreviations: CAL, calcium‐acetate‐lactate; CAT, calcium chloride and diethylentriaminepentaacetic acid; F‐AAS, flame atomic absorption spectroscopy; ICP‐OES, inductively coupled plasma optical emission spectroscopy.

^a^ Dew point and relative air humidity were not recorded plot‐wise but with three loggers per forest type so that minimum and maximum equal the average. These variables were just used for a comparison between forest types and were not included in within‐forest comparisons and statistical analyses.

Furthermore, to detect variation in the substrate (henceforth referred to as “soil”), we took mixed samples of both litter and the organic layer, consisting of four soil cores from the edges of each plot, thereby minimizing within‐plot disturbance. Soil samples were dried at 40°C prior to sieving (< 2 mm) for further extraction steps and finely ground for combustion analyses. Total nitrogen (N) and carbon (C) were determined via combustion (Vario Max Cube, Elementar, Germany). Different extraction methods for plant‐available soil nutrients were used to determine magnesium (Mg), phosphorus (P), potassium (K), calcium (Ca), and manganese (Mn), which are provided in Table 3. Spectrometric analyses were performed to detect Ca and Mn by inductively coupled plasma optical emission spectroscopy (Spectro Arcos, Spectro analytical Instruments GmbH, Germany), and K and Mg by flame atomic absorption spectroscopy (iCE 3000 series; Thermo Fisher Scientific, USA). Photometric analysis was used to determine P in a flow injection analyzer (QuickChem 8500 series 2; Lachat Instruments, USA). Soil pH was measured in a water extract. All used methods accomplished the analytical requirements and standards according to DIN EN 17025:2018‐03.

### 2.4 Data Analysis

**FIGURE A1:**
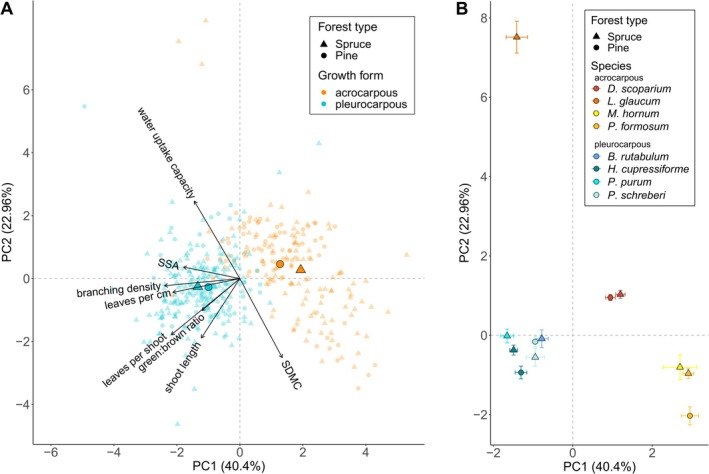
Principal component analysis (PCA) on log‐transformed and scaled functional traits based on all observations by excluding cushion depth and *F*_v_/*F*_m_, showing (A) individual traits (small dots) with superimposed centroids for growth form and forest type (big dots), and (B) centroids ± standard error of each species, separated by growth form and forest type. Colour and shape coding applies as indicated in the legends. SDMC, shoot dry matter content; SSA, specific shoot area.

We used a Principal Component Analysis (PCA) to assess the variation in the overall trait dataset on log‐transformed, centered, and scaled data. We used the R package *vegan* (Oksanen et al. 2024) to superimpose significantly fitted variables (*p* < 0.05) based on a permutation test. Due to missing trait data for some species resulting from the sampling (mainly for cushion depth and *F*_v_/*F*_m_), nearly 150 out of 427 observations were removed during the analysis. However, a PCA based on all observations excluding the abovementioned traits showed a nearly identical pattern with the main difference that the forest types were even more similar in trait composition (Figure A1).

**FIGURE 2:**
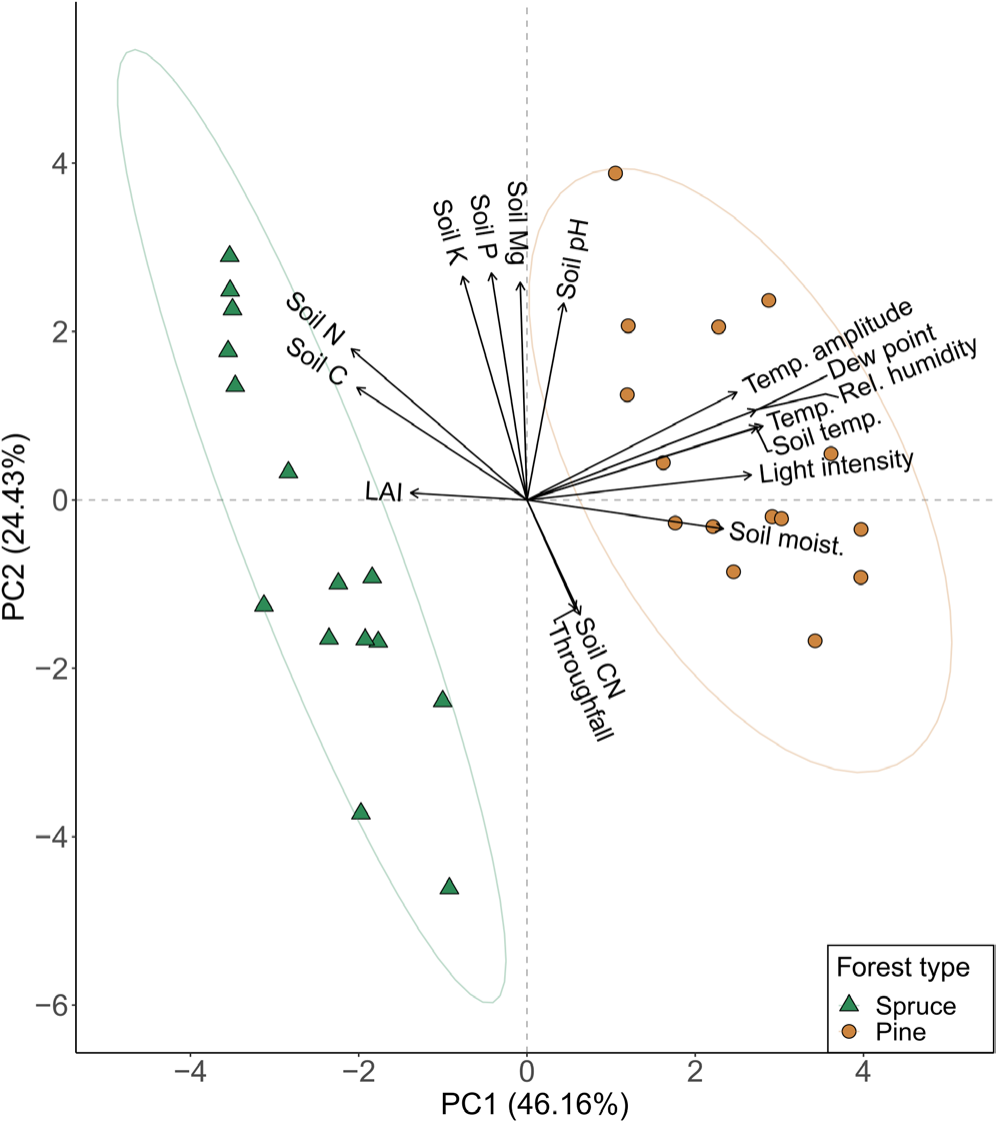
Principal Component Analysis of measured centred and scaled environmental variables for the two forest types spruce and pine. The ellipses represent the 95% confidence area. LAI, leaf area index; rel. humidity, relative humidity; soil moist., soil moisture; temp., temperature.

As a PCA on environmental conditions showed that forest types differed strongly (see separation following axis 1 of Figure 2), and we were interested in small‐scale responses, the following analyses were performed separately for each forest type.

**FIGURE A2:**
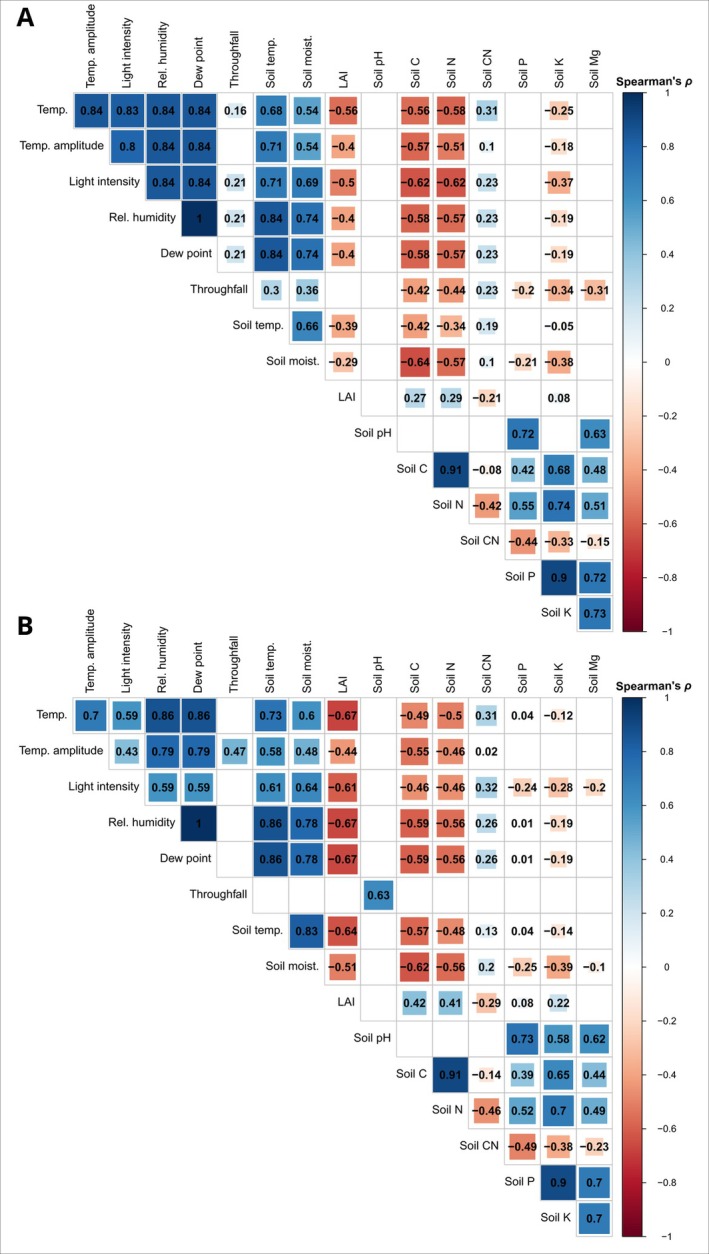
Spearman rank correlation plots with superimposed significant (*p* < 0.05) correlation coefficient *ρ* across all environmental variables (A) as yearly average for all models but *F*_v_/*F*_m_, and (B) as two‐week average before fluorescence measurements for the *F*_v_/*F*_m_ models as explained in Section 2. Blue tiles indicates a positive, and red tiles a negative correlation. For the mixed effects models, we removed all collinear variables with *ρ* ≥ |0.7|, namely light intensity, temperature amplitude, relative humidity, dew point, soil C, P, and K content. The same variables and soil temperature but not light intensity were removed for the *F*_v_/*F*_m_ models. Abbreviations are as in Figure 2.

To assess whether functional traits are related to small‐scale environmental variation, we ran (generalised) linear mixed effect models for every functional trait using the R package *glmmTMB* (Brooks et al. 2017). The model structure was “Trait ~ (env_1_ + env_2_ + …) × growth form + (1|random effect)” where “env” was a measured environmental variable (see Table 3 for all variables), and “random effect” was either “species”, “site:repetition”, or both. The most adequate random effect was chosen in an AIC model comparison following Zuur et al. (2009). To avoid collinearity, we selected one of two correlating variables with *ρ ≥* |0.7| (Spearman rank correlation) based on ecological importance (full correlation plots and information on removed variables are provided in Figure A2). For *F*_v_/*F*_m_, we used the two‐week average of environmental conditions before measurements, instead of the overall average, as this trait is strongly influenced by current weather conditions in bryophytes due to their poikilohydric nature. All independent variables were standardised to (0, 1) to allow for estimates of relative importance between them.

To meet the model assumptions, we log‐transformed leaves per shoot, green:brown ratio, cushion depth, water uptake capacity, and SSA, and removed extreme outliers after visual inspection in the spruce forest models. In the pine forest models, we log‐transformed leaves per cm, leaves per shoot, green:brown ratio, SDMC, and SSA. For branching density, we excluded acrocarpous mosses as they do not usually branch and would zero‐inflate the model. Minimum adequate models were obtained using backward model selection (Zuur et al. 2009). To test if the model estimates differed from zero, we used the R package *emmeans* (Lenth 2023). For selected traits and the most important environmental variables, we performed analyses of covariance (ANVOCAs) with “species” as interaction term.

All data analysis was carried out in R v. 4.2.3 using the packages *tidyverse* (Wickham et al. 2019), *reshape2* (Wickham 2007), and *lubridate* (Grolemund and Wickham 2011) for general data preparation.

## 3 Results

### 3.1 Functional Trait Composition and Coordination

**FIGURE 3:**
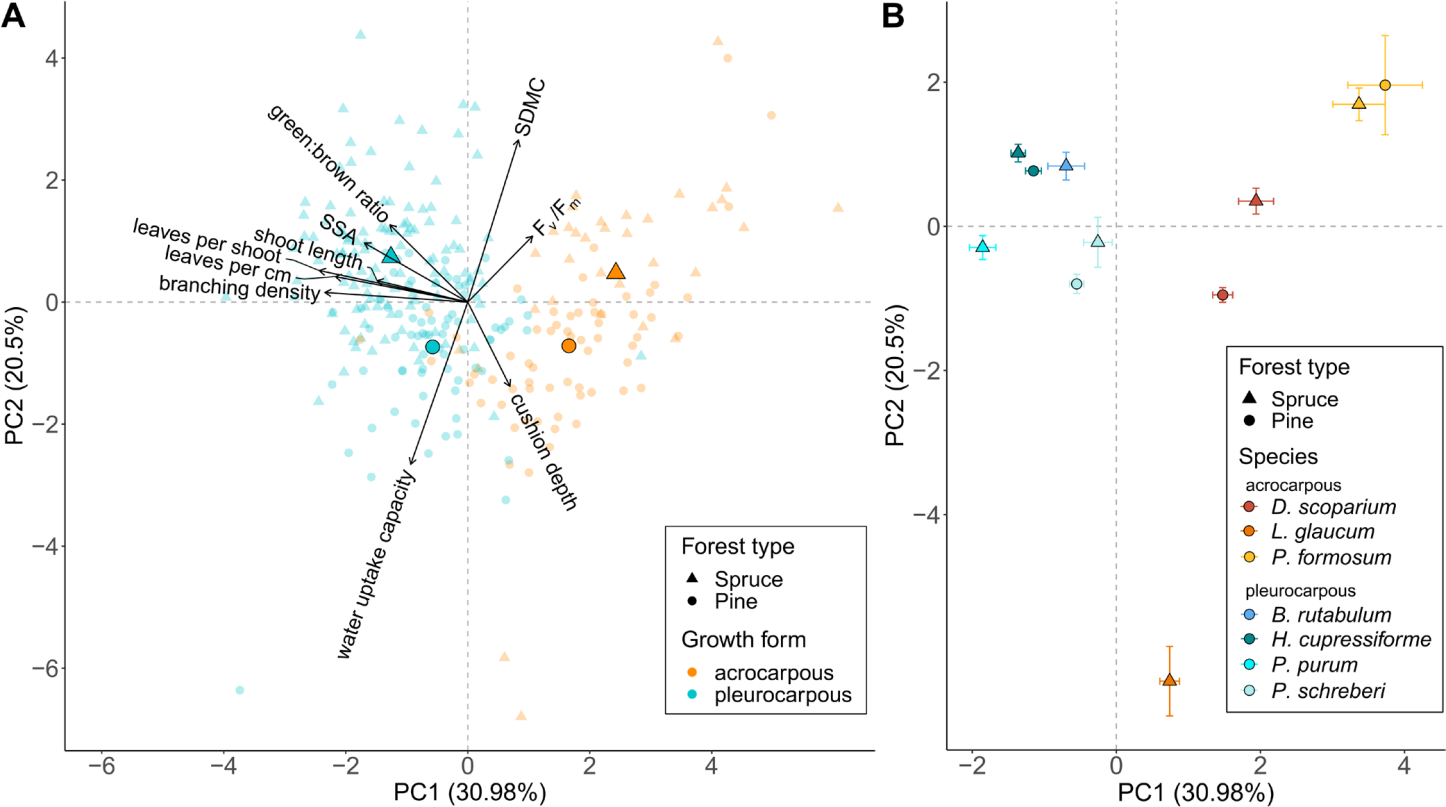
Principal component analysis of log‐transformed, centred and scaled functional traits showing (A) individual traits (small dots) with superimposed centroids for growth form and forest type (big dots), and (B) centroids ± standard error of each species, separated by growth form and forest type. Colour and shape coding applies as indicated in the legends, warm colours represent acrocarpous and cold colours represent pleurocarpous mosses. Due to missing trait data in some species, they were automatically excluded from the analysis, resulting in a reduced number of observations. *F*_v_/*F*_m_, photosynthetic efficiency; SDMC, shoot dry matter content; SSA, specific shoot area.

The PCA on functional traits revealed a morphological gradient on the first and a physiological gradient on the second axis (Figure 3A). The first axis (PC1: 40.4% explained variance) was positively linked to the morphological traits shoot length, leaves per cm, and branching density, concomitant to leaves per shoot, green:brown ratio, and specific shoot area (SSA). The second axis (PC2: 22.96% explained variance) was negatively associated with the physiological traits water uptake capacity and positively associated with shoot dry matter content (SDMC) and photosynthesis efficiency (*F*_v_/*F*_m_). The traits were further clearly segregated by growth form along PC1, forming two distinct groups of acro‐ and pleurocarpous mosses. In contrast, the forest type showed a less strong influence on trait composition along PC2 and was related to differences in physiological traits. Additional statistical comparisons between growth form and forest type for each trait also showed differences between growth forms but not between forest types (Table A1).

On the species level, largest differences were observed across acrocarpous species along PC2, with *L. glaucum* exhibiting a very high water uptake capacity (WUC) at one extreme, and *Polytrichum formosum* displaying a high SDMC at the other extreme (Figure 3B). The centroids of pleurocarpous species were more clumped together, although overall variation was comparable to acrocarpous mosses.

### 3.2 Links Depending on Growth Form and Forest Type

**FIGURE 4:**
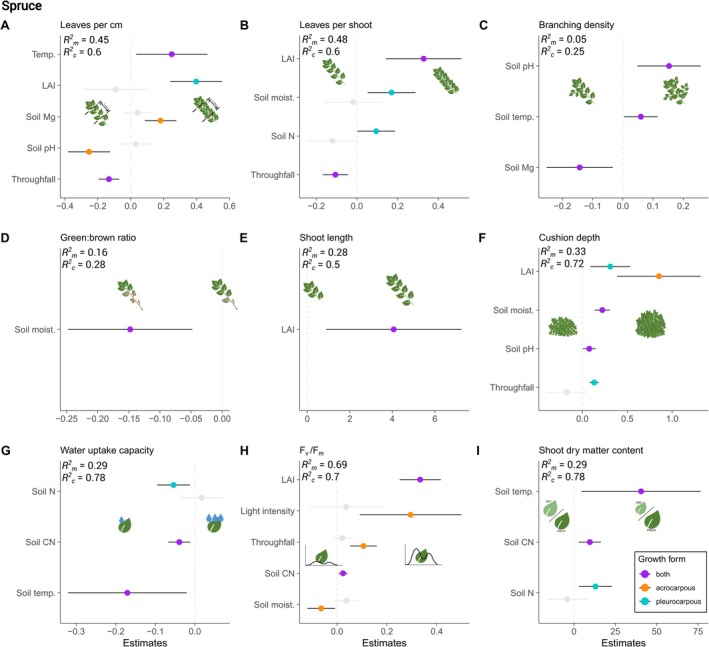
Responses of moss functional traits to small‐scale environmental variability across all species in the spruce forest. Shown are the model estimates that were significantly different from zero, based on mixed effects models and corresponding marginal ($R_{m}^{2}$) and conditional ($R_{c}^{2}$) *R* ^2^ as described in Section 2. The models for B, D, F, and G were log‐transformed to meet the model assumptions. The model for specific shoot area did not have any significant predictor and is therefore not shown, but details are provided in Table A1. The color code for the growth forms applies as indicated in the legend. Greyed‐out points represent non‐significant estimates when growth forms differed in their responses.

**FIGURE 5:**
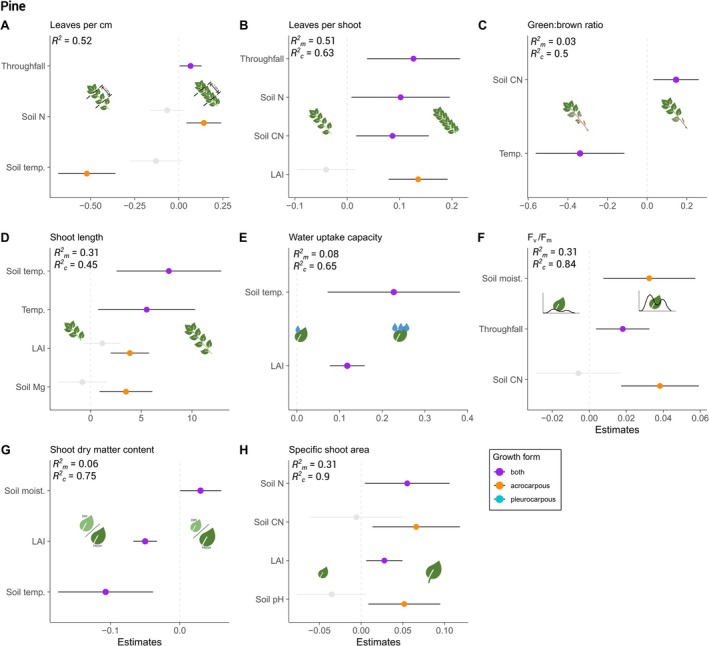
Responses of moss functional traits to small‐scale environmental variability across all species in the pine forest. Shown are the model estimates that were significantly different from zero, based on mixed effects models and corresponding marginal ($R_{m}^{2}$) and conditional ($R_{c}^{2}$) *R* ^2^ as described in Section 2. The models for A–C, G, and H were log‐transformed to meet the model assumptions. Models for branching density and cushion depth did not have any significant predictor and are therefore not shown, but details are provided in Table A2. For A, the best model did not include a random effect, which is why *R* ^2^ is shown. The color code for the growth forms applies as indicated in the legend. Greyed out points represent non‐significant estimates when growth forms differed in their responses.

Results from the mixed effects models showed specific responses to small‐scale environmental variation in both spruce and pine forests (Figures 4 and 5), resulting in many ambiguous relationships with an increase in water, light, nutrient availability (Table 2). Detailed model outputs and statistics are provided in Tables A2 and A3. While in the spruce forest, an increase in throughfall led to a decrease in leaves per cm and leaves per shoot (Figure 4A,B), mosses in the pine forest responded with an increase (Figure 5A,B). While SSA and shoot length did not show any or only a low response in the spruce forest, they showed a higher response in the pine forest. In addition, we also found growth‐form‐specific responses. For example, in the spruce forest, photosynthetic efficiency (*F*_v_/*F*_m_) responded to light intensity, throughfall, and soil moisture only in acrocarpous mosses, while leaves per shoot responded to soil moisture and soil N only in pleurocarpous mosses (Figure 4B,F). Yet, overall, most responses were similar between growth forms.

### 3.3 Link to Water Availability

In the spruce forest, increasing throughfall led to a decreased number of leaves and lower *F*_v_/*F*_m_ for acrocarpous mosses; cushion depth slightly increased for pleurocarpous mosses (Figure 4A,B,F,H). Despite its positive correlation with throughfall (Spearman rank correlation, *ρ* = 0.37, *p* < 0.001; Figure A2), the response to soil moisture was different and partly opposite, leading to ambiguous or contrary relationships to those predicted with increasing water availability (Table 2). With increasing soil moisture, leaves per shoot also increased in pleurocarpous mosses, and cushions were thicker, while *F*_v_/*F*_m_ in acrocarpous mosses and the green:brown ratio in both growth forms decreased (Figure 4B,D,F,H). In the pine forest, increased throughfall generally led to more leaves and higher *F*_v_/*F*_m_, while higher soil moisture increased SDMC and *F*_v_/*F*_m_ in acrocarpous mosses (Figure 5A,B,F,G).

### 3.4 Link to Light Availability

Light availability, that is, here foremost characterised by LAI of the aboveground canopy being strongly correlated with light intensity (*ρ* = −0.51, *p* < 0.001; Figure A2), was an overall important environmental variable showing mostly positive relationships with the traits: with increasing LAI, that is, less light available, mosses in the spruce forest generally tended to have more leaves, longer shoots, thicker cushions, and higher *F*_v_/*F*_m_ values (Figure 4A,B,E,F,H). Increased light intensity led to an increase in *F*_v_/*F*_m_ in acrocarpous mosses only (Figure 4H). In the pine forest, with increasing LAI, mosses tended to have higher WUC and SSA, more leaves per shoot, and longer shoots in acrocarpous mosses, and generally a lower SDMC (Figure 5B,D,E,G,H).

### 3.5 Link to Soil Nutrient Availability and pH

The trait responses to soil nutrients and pH were generally more ambiguous than expected. In the spruce forest, an increase in soil CN ratio led to overall higher SDMC and *F*_v_/*F*_m_ values but lower WUC (Figure 4G–I), whereas soil N only influenced pleurocarpous mosses, which showed more leaves per shoot, higher SDMC, and lower WUC values with increasing N (Figure 4B,G,I). A higher soil pH led to mosses with a higher branching density, thicker cushions, but fewer leaves per cm in acrocarpous mosses (Figure 4A,C,F). In the pine forest, an increased soil CN ratio led to more leaves per shoot and a higher green:brown ratio, as well as a higher SSA and *F*_v_/*F*_m_ in acrocarpous mosses (Figure 5B,C,F,H). The response to soil N was similar, with mosses showing generally more leaves and a higher SSA (Figure 5A,B,H). Soil pH was less important than in the spruce forest, only increasing the SSA of acrocarpous mosses at higher pH (Figure 5H).

**FIGURE 6:**
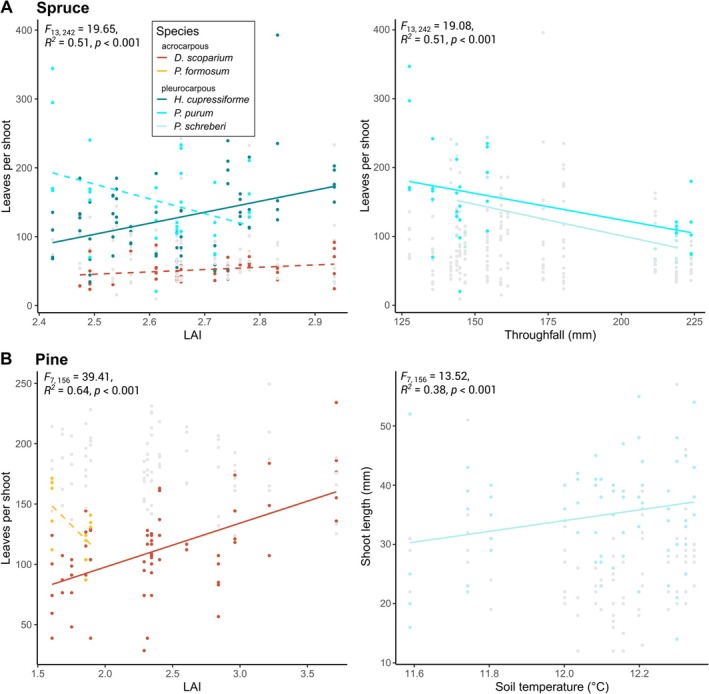
Selected responses of leaves per green and shoot length to important environmental variables for (A) spruce, and (B) pine forest types, based on ANCOVAs as described in Section 2. Solid lines represent significant responses (*p* < 0.05), that is, the slopes were significantly different from zero, while dashed lines indicate marginally significant responses (*p* < 0.1). The colour code applies as indicated in the legend with warm colours representing acrocarpous and cold colours representing pleurocarpous species. Data of non‐significant species are greyed out.

**FIGURE A3:**
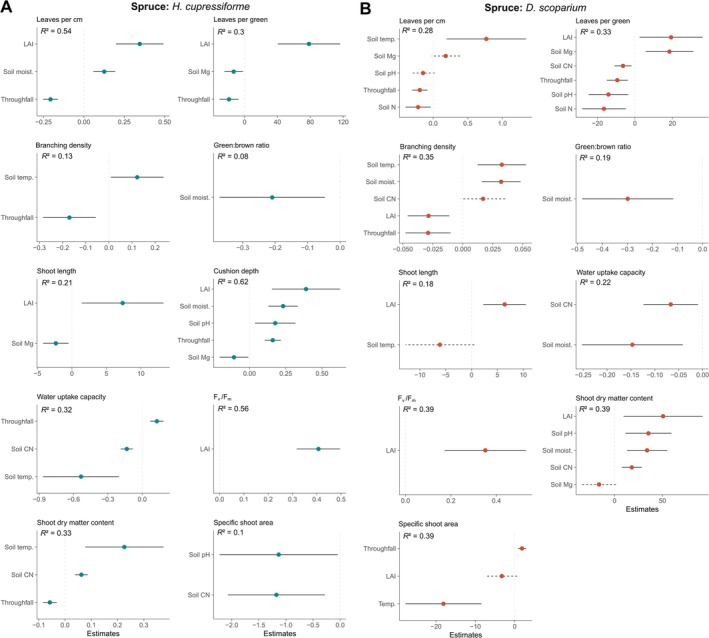
Coefficient plots for intraspecific trait responses based on species‐wise linear models for the two most dominant species in the spruce forest, namely (A) *Hypnum cupressiforme* , and (B) *Dicranum scoparium* . The panels show the goodness of fit *R* ^2^ for each model. Dashed lines indicate marginally significant estimates (*p* < 0.1), while solid lines indicate significant differences from zero (*p* < 0.05).

**FIGURE A4:**
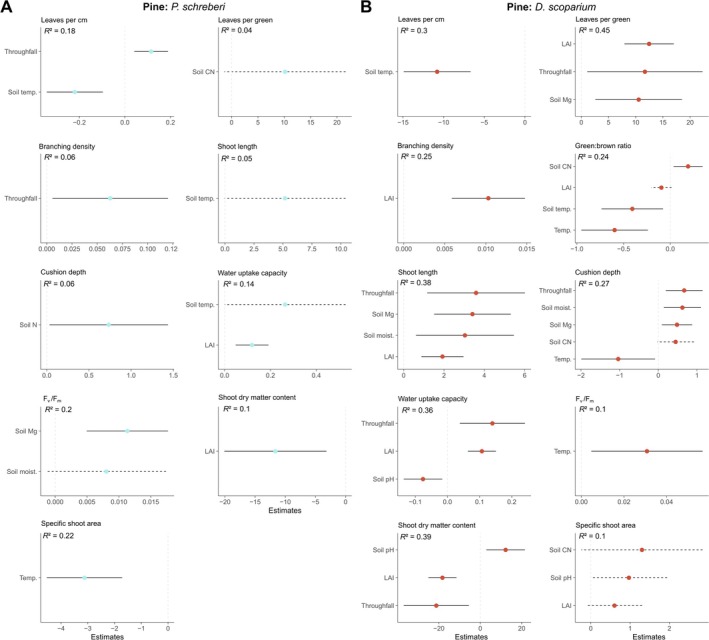
Coefficient plots for intraspecific trait responses based on species‐wise linear models for the two most dominant species in the pine forest, namely (A) *Pleurozium schreberi* , and (B) *Dicranum scoparium* . The panels show the goodness of fit *R* ^2^ for each model. Dashed lines indicate marginally significant estimates (*p* < 0.1), while solid lines indicate significant differences from zero (*p* < 0.05).

Additional ANCOVAs on leaves per shoot and LAI (spruce: *F*_13,242_ = 19.65, *R* ^2^ = 0.51, *p* < 0.001; pine: *F*_7,156_ = 39.41, *R* ^2^ = 0.64, *p* < 0.001), leaves per green and throughfall (spruce: *F*_13,242_ = 19.08, *R* ^2^ = 0.51, *p* < 0.001), and shoot length and soil temperature (pine: *F*_7,156_ = 13.52, *R* ^2^ = 0.38, *p* < 0.001), respectively, showed that certain species drove the main pattern, mainly being the dominating mosses occurring along the whole captured variability, namely *Hypnum cupressiforme* and *Dicranum scoparium* in the spruce forest, and *Pleurozium schreberi* and *D. scoparium* in the pine forest (Figure 6). Additional analyses of species‐specific responses for those species revealed intraspecific variation in both forest types (see Figures A3 and A4): *H. cupressiforme* and *D. scoparium* responded strongly, while *P. schreberi* was less sensitive, showing species‐specific responses for most measured traits.

## 4 Discussion

This study clearly shows that both the moss community and individual species studied here were functionally highly sensitive to small‐scale environmental variability within and between temperate coniferous forest types. Further, we showed that the selected traits responded along a morphological and a physiological axis. Growth forms, that is, pleuro‐ and acrocarpous mosses, were strongly distinct in their trait composition, which was linked to morphological features, while the forest type was of minor importance. Long‐term processes certainly shape the community composition under specific environmental variability, and found patterns are, to a certain extent, subject to species turnover. Yet, we also found intraspecific variation indicating strong species‐specific plasticity along small‐scale environmental variability, as the main pattern was mainly driven by species occurring along the full range of captured variability, that is, community dominating species.

### 4.1 Functional Differences in Growth Form and Forest Type

As has been shown for traits of vascular plant species (Deilmann, Ulrich, et al. 2024; Lienin and Kleyer 2012), also in mosses some traits were more responsive than others to the investigated environmental variability, which was mainly forest‐type‐ and growth‐form‐specific. This pattern aligns with previous studies showing varying trait compositions across different habitats and species (Grau‐Andrés et al. 2022), indicating that certain traits may be more useful than others depending on the context.

Similar to vascular plants, also in bryophytes the economic spectrum can be applied showing strategies of rapid resource acquisition on the one hand and conservation of resources on the other hand (Grau‐Andrés et al. 2022; Wang et al. 2017), such as acrocarpous mosses occurring in rather drier and warmer places. Consequently, we would assume growth‐form‐specific responses to environmental variability, which, as we show here, happen already at the small scale. We therefore can confirm our first hypothesis that trait composition differs between growth forms and forest types even though the former was much more pronounced in our study.

The grouping of functional traits along a morphological and a physiological axis is known from vascular plants (Díaz et al. 2016) and is also true for the investigated bryophyte traits here. In our case, morphological trait values mainly reflected the growth form and associated life strategies. In the current study, two main strategies were compared: light scavenging pleurocarpous mosses that are mostly prostrate growing and frequently branched, preferring shady and humid habitats, versus efficient water‐using acrocarpous mosses that mostly grow erect and form dense cushions or turfs, tolerating more sun‐exposed and xeric habitats (Bates 1998). Our results suggest that pleurocarpous mosses can be well described by the morphological traits branching density, leaves per cm, and leaves per shoot, shoot length, and green:brown ratio, which usually showed higher values than in acrocarpous mosses. This suggests that it may be useful to include growth forms in functional trait studies, as they provide additional information. So far, however, most studies exploring moss functional traits differentiated between species (e.g., Grau‐Andrés et al. 2022; Wang et al. 2014; Waite and Sack 2010) but not many studies have included growth form as a functional group, even though they may increase functional resolution, especially when species determination is a challenge (Lett et al. 2022).

### 4.2 Link to Water Availability

One of the most important drivers of trait responses is water availability, here characterised by precipitation throughfall and soil moisture. This is not surprising when considering the poikilohydry of bryophytes, which is strongly controlled by the immediate external environmental conditions, particularly water (Glime 2022; Proctor 2008). Growing at places where more throughfall, and hence nutrients are available will, thus, provide better growing conditions (Bates 2008). Consequently, this leads to trait responses such as an altered number of leaves, longer shoots, or increased photosynthetic efficiency. In fact, growth is only possible if sufficient water is provided, regardless of temperature, light, or nutrient supply, as was shown for *P. schreberi* (Longton and Greene 1979).

However, our findings were forest type‐specific, and the responses to throughfall and soil moisture were different, often even opposite. This is why our hypothesized responses to an increase in water availability were only partly supported by the actual measurements. Furthermore, this ambiguity shows that mosses play an important role in rainfall interception and retention (Porada et al. 2018), which is further supported by the surprisingly weak positive correlation of throughfall and soil moisture in our study. Forest type‐specific differences likely were the result of species‐specific responses, as the two dominating species per each forest type, namely *H. cupressiforme* and *P. schreberi* , often showed opposite responses to water availability.

Contrary to our expectations, branching density did not respond to changes in water availability, that is, neither to throughfall nor to soil moisture. In the species‐specific models, we found responses, but the models did not explain much of the variance. This suggests that branching density is either a rather insensitive and conserved trait not being strongly related to external water storage or that our small‐scale variability was too small to capture responses in this trait. As there is no literature investigating branching density or its response to a larger environmental variability, this leaves a knowledge gap for future studies.

Overall, our second hypothesis that morphological microstructures, such as leaves per cm, leaves per shoot, or branching density, to capture extra capillary water, decrease with increasing water availability can partly be confirmed. Further research on quantifying the role that morphological traits play for external water storage is needed.

### 4.3 Link to Light Availability

Trait values that relate to overall leaf surface, such as shoot length and leaves per cm of shoot, as well as photosynthetic efficiency responded well to varying light availability. More specifically, a higher LAI of aboveground canopy, and thus, lower light availability at the forest floor, led to longer shoots with more leaves, deeper cushions, and higher fluorescence. Higher shading by, for example, a denser tree cover, also alleviates temperature and thus reduces evapotranspiration of the understory (De Frenne et al. 2021), creating a more favorable microclimate for ground‐dwelling forest mosses. This, in turn, promotes growth by bridging periods without rainfall (Proctor 2008), mitigating light and temperature excesses, and thus alleviating photoinhibition, to which forest‐dwelling shade‐tolerant moss species in particular are susceptible (Marschall and Proctor 2004; Proctor and Bates 2018). The overall response was mainly driven by species that occurred along the whole captured variability, here mainly *H. cupressiforme* , *D. scoparium* , and *P. schreberi* , indicating that species‐specific plasticity was a driving force (see Figures A3 and A4). Overall, as the response to LAI showed a strong and consistent pattern, we can accept our third hypothesis that trait values related to photosynthetic efficiency and leaf surface overall decline with increasing light availability.

### 4.4 Link to Soil Nutrient Availability and pH

The responses of traits to soil nutrient availability and pH were more ambiguous (Table 2). On the one hand, increasing soil pH, here being strongly positively correlated to plant available nutrients (P, K, Mg; Figure A2), led to higher branching density and thicker cushions, supporting our hypothesis that soil nutrition also promoted the growth of bryophytes, not only vascular plants, and is in line with previous findings (e.g., Ayres et al. 2006). On the other hand, lower pH increased leaves per cm; that is, less available nutrients were linked to more leaves per cm. Furthermore, increasing soil CN ratio, that is, increasing N limitation, led to an increase in SDMC, meaning an investment in structural biomass and photosynthetic efficiency (*F*_v_/*F*_m_), hence challenging our hypothesis. However, the causal relationship remains unclear: the observed link between traits and soil nutrients could be attributed to an upward movement of minerals (Glime 2022). Alternatively, the moss layer could promote mineralisation by regulating soil moisture and temperature (Gall et al. 2024; Gornall et al. 2007; Turetsky 2003). Usually, bryophytes derive nutrients from dry and wet deposition (Bates 2008), but also direct uptake from the soil of nitrogen (Ayres et al. 2006), phosphorus and calcium (Bates 1992) were reported, and even deeper soil may provide bryophytes with nutrients by a combination of concentration gradient and capillary action (Glime 2022). It seems that the nutrient supply from the substrate could be more important than previously thought, and the causal relationship needs to be disentangled in experimental studies.

## 5 Conclusion

We here show that both bryophyte communities (interspecific) and single species (intraspecific) respond highly sensitively to small‐scale environmental variability and that functional traits are a useful tool to capture those responses. Forest type appears to be less important than growth form when comparing trait composition, making growth form a useful approach in bryophyte response studies. Certain causal relationships between trait‐ and environmental variability, particularly regarding soil nutrients and pH, are still unclear and need to be disentangled in future experimental studies. Overall, bryophyte functional traits have great potential to reveal even small changes in the environment and therefore should be more specifically included in response studies.

## Author Contributions

**T. J. Deilmann:** conceptualization (equal), data curation (lead), formal analysis (lead), investigation (lead), methodology (equal), project administration (equal), visualization (lead), writing – original draft (lead), writing – review and editing (lead). **M. Bernhardt‐Römermann:** conceptualization (equal), methodology (equal), writing – review and editing (equal). **J. Hentschel:** conceptualization (equal), methodology (equal), writing – review and editing (supporting). **P. Gros:** investigation (supporting), writing – review and editing (supporting). **C. Römermann:** conceptualization (equal), funding acquisition (lead), methodology (equal), resources (lead), supervision (lead), writing – review and editing (equal).

## Conflicts of Interest

The authors declare no conflicts of interest.

## Data Availability

All data and code used in this study can be retrieved from Zenodo (https://doi.org/10.5281/zenodo.15689047).

## Appendix A

**TABLE A1:** Averaged species means for recorded functional traits per forest type ± respective standard error. Missing values or standard errors can result from low sample size. Statistical differences are based on species means using either Welch two sample *t*‐test or Wilcoxon rank sum test, depending on the data distribution.

Forest type	Species	Growth form	Leaves per cm	Leaves per shoot	Branching density (cm^−1^)	Green:brown ratio	Shoot length (mm)	Cushion depth (cm)	WUC (g g^−1^)	*F*_v_/*F*_m_	SDMC (mg g^−1^)	SSA (mm^2^ mg^−1^)
Differences between forest types			No	No	No	No	No	No	No	No	No	No
Differences between growth forms			No	Yes	Yes	Yes	Yes	No	No	Yes	No	No
Spruce	*D. scoparium*	A	27.84 ± 1.25	51.68 ± 2.44	0.04 ± 0.01	1.67 ± 0.1	19.18 ± 0.66	3.34 ± 0.21	1.39 ± 0.04	0.66 ± 0.03	423.07 ± 6.61	22.09 ± 0.69
Spruce	*L. glaucum*	A	51.33 ± 8.35	33 ± 4.04	0.15 ± 0.01	0.57 ± 0.22	6.67 ± 0.67	4 ± 0	5.44 ± 0.21	0.78 ± 0.01	155.49 ± 5.04	23.28 ± 2.46
Spruce	*M. hornum*	A	36.8 ± 2.24	38.6 ± 2.48	0	1.78 ± 0.27	10.8 ± 1.16	—	0.57 ± 0.09	—	645.34 ± 37.76	29.58 ± 1.68
Spruce	*P. formosum*	A	27.4 ± 1.42	60.49 ± 3.42	0	1.01 ± 0.07	22.47 ± 0.82	3.5 ± 0.37	0.74 ± 0.02	0.73 ± 0.03	580.75 ± 8.46	13.2 ± 0.31
Spruce	*B. rutabulum*	P	41.1 ± 1.68	96.68 ± 7.13	0.38 ± 0.06	2.8 ± 0.31	23.55 ± 1.31	1.74 ± 0.13	1.23 ± 0.04	0.45 ± 0.01	453.64 ± 9.7	29.79 ± 0.64
Spruce	*H. cupressiforme*	P	46.45 ± 1.36	127.67 ± 6.64	0.56 ± 0.03	2.3 ± 0.27	27.55 ± 1.06	2.08 ± 0.1	1.25 ± 0.03	0.5 ± 0.02	452.04 ± 7.05	35.42 ± 0.49
Spruce	*P. purum*	P	41.66 ± 1.12	151.66 ± 10.98	0.77 ± 0.16	2.05 ± 0.17	35.86 ± 2	2.77 ± 0.21	1.61 ± 0.05	0.44 ± 0.02	388.58 ± 6.89	20 ± 0.39
Spruce	*P. schreberi*	P	34.59 ± 3.13	122.94 ± 13.84	0.48 ± 0.03	3.24 ± 0.7	36.24 ± 3.06	4.14 ± 0.37	1.31 ± 0.05	0.43 ± 0.01	436.09 ± 10.12	20.94 ± 0.49
Pine	*D. scoparium*	A	29.06 ± 0.87	68.38 ± 3.6	0.01 ± 0	1.21 ± 0.06	23.42 ± 0.77	4.51 ± 0.15	1.47 ± 0.03	0.45 ± 0.01	409.68 ± 5.36	20.09 ± 0.45
Pine	*L. glaucum*	A	52 ± 2	59 ± 11	0	0.86 ± 0.22	11.5 ± 2.5	—	6.38 ± 0.9	0.76	137.47 ± 16.69	25.36 ± 2.58
Pine	*P. formosum*	A	27.85 ± 2.55	79.08 ± 5.72	0	0.81 ± 0.09	29.69 ± 1.61	6.4 ± 0.4	0.59 ± 0.04	0.65 ± 0.07	635.16 ± 18.25	12.11 ± 0.28
Pine	*H. cupressiforme*	P	36.5 ± 2.68	121.17 ± 7.54	0.45 ± 0.05	2.7 ± 0.41	34.33 ± 2.5	1.67 ± 0.17	1.13 ± 0.04	0.41 ± 0.01	471.79 ± 8.32	36.17 ± 2.82
Pine	*P. schreberi*	P	38.77 ± 0.9	132.1 ± 4.15	0.49 ± 0.02	1.12 ± 0.07	34.82 ± 1.02	5.96 ± 0.21	1.45 ± 0.08	0.42 ± 0	423.07 ± 7.39	20.26 ± 0.47

Abbreviations: A, acrocarpous; P, pleurocarpous. For other abbreviations, see Table 2.

**TABLE A2:** Model outputs of all mixed effects models for the spruce forest type with respective marginal *R* ^2^ ($R_{m}^{2}$) and conditional *R* ^2^ ($R_{c}^{2}$). Presented are the estimates with standard error. Significant estimates are marked in bold. “—” represents that the variable or interaction was not present in the minimum adequate model. The estimated marginal means of the coefficient plots are based on the model estimates, but estimates may differ.

	Spruce	Leaves per cm	Leaves per shoot	Shoot length	Green:brown ratio	Branching density	Cushion depth	Shoot dry matter content	Specific shoot area	Water uptake capacity	*F*_v_/*F*_m_
	Explanatory variable	$R_{m}^{2}$ = 0.45, $R_{c}^{2}$ = 0.6	$R_{m}^{2}$ = 0.48, $R_{c}^{2}$ = 0.6	$R_{m}^{2}$ = 0.28, $R_{c}^{2}$ = 0.5	$R_{m}^{2}$ = 0.16, $R_{c}^{2}$ = 0.28	$R_{m}^{2}$ = 0.05, $R_{c}^{2}$ = 0.25	$R_{m}^{2}$ = 0.33, $R_{c}^{2}$ = 0.72	$R_{m}^{2}$ = 0.29, $R_{c}^{2}$ = 0.78	$R_{m}^{2}$ = 0, $R_{c}^{2}$ = 0.82	$R_{m}^{2}$ = 0.29, $R_{c}^{2}$ = 0.78	$R_{m}^{2}$ = 0.69, $R_{c}^{2}$ = 0.7
	(Intercept)	**3.53 ± 0.13**	**4.11 ± 0.26**	**16.7 ± 3.49**	0.2 ± 0.15	**0.5 ± 0.07**	**0.83 ± 0.28**	**581.6 ± 47.85**	3.14 ± 0.13	**−0.35 ± 0.2**	**0.65 ± 0.07**
Main effect	LAI	−0.09 ± 0.1	**0.33 ± 0.09**	**4.06 ± 1.62**	—	—	**0.85 ± 0.24**	—	—	—	**0.33 ± 0.04**
	Light intensity	—	—	—	—	—	—	—	—	—	**0.29 ± 0.1**
	Soil CN	—	—	—	—	—	—	**9.59 ± 3.39**	—	**−0.04 ± 0.01**	**0.02 ± 0.01**
	Soil Mg	**0.18 ± 0.05**	—	—	—	**−0.14 ± 0.06**	—	—	—	—	—
	Soil N	—	−0.12 ± 0.06	—	—	—	—	−4.06 ± 6.35	—	0.02 ± 0.03	−0.02 ± 0.02
	Soil moist.	−0.03 ± 0.06	−0.02 ± 0.07	—	**−0.15 ± 0.05**	—	**0.22 ± 0.04**	—	—	—	**−0.06 ± 0.03**
	Soil pH	**−0.25 ± 0.07**	—	—	—	**0.15 ± 0.05**	**0.08 ± 0.04**	—	—	—	**−0.07 ± 0.02**
	Soil temp.	—	0.44 ± 0.27	—	—	**0.06 ± 0.03**	—	**40.57 ± 18.31**	—	**−0.17 ± 0.08**	—
	Temp.	**0.25 ± 0.11**	—	—	—	—	—	—	—	—	—
	Throughfall	**−0.13 ± 0.03**	**−0.11 ± 0.03**	—	—	—	−0.17 ± 0.11	—	—	—	**0.11 ± 0.03**
	GF_pleurocarpous	**0.32 ± 0.09**	0.36 ± 0.3	**12.86 ± 4.48**	**0.4 ± 0.19**	—	0.22 ± 0.32	**−124.62 ± 60.01**	—	**0.52 ± 0.25**	**−0.31 ± 0.08**
Interaction	LAI:GF	**0.49 ± 0.12**	—	—	—	—	**−0.54 ± 0.25**	—	—	—	—
	Light intensity:GF	—	—	—	—	—	—	—	—	—	**−0.26 ± 0.12**
	Soil Mg:GF	**−0.14 ± 0.05**	—	—	—	—	—	—	—	—	—
	Soil N:GF	—	**0.21 ± 0.07**	—	—	—	—	**17.03 ± 7.5**	—	**−0.07 ± 0.03**	**0.05 ± 0.02**
	Soil moist.:GF	0.1 ± 0.05	**0.19 ± 0.09**	—	—	—	—	—	—	—	**0.1 ± 0.03**
	Soil pH:GF	**0.28 ± 0.07**	—	—	—	—	—	—	—	—	**0.07 ± 0.03**
	Soil temp.:GF	—	**−0.72 ± 0.31**	—	—	—	—	—	—	—	—
	Throughfall:GF	—	—	—	—	—	**0.31 ± 0.12**	—	—	—	**−0.08 ± 0.03**

Abbreviations: GF, growth form (acrocarpous and pleurocarpous); LAI, leaf area index; Soil moist., soil moisture; Temp., temperature.

**TABLE A3:** Model outputs of all mixed effects models for the pine forest type with respective marginal *R* ^2^ ($R_{m}^{2}$) and conditional *R* ^2^ ($R_{c}^{2}$). Presented are the estimates with standard error. Significant estimates are marked in bold. “—” represents that the variable or interaction was not present in the minimum adequate model. The estimated marginal means of the coefficient plots are based on the model estimates, but estimates may differ.

	Pine	Leaves per cm	Leaves per shoot	Shoot length	Green:brown ratio	Branching density	Cushion depth	Shoot dry matter content	Specific shoot area	Water uptake capacity	*F*_v_/*F*_m_
	Explanatory variable	*R* ^2^ = 0.52	$R_{m}^{2}$ = 0.51, $R_{c}^{2}$ = 0.63	$R_{m}^{2}$ = 0.31, $R_{c}^{2}$ = 0.45	$R_{m}^{2}$ = 0.03, $R_{c}^{2}$ = 0.5	$R_{m}^{2}$ = 0.05, $R_{c}^{2}$ = 0.25	$R_{m}^{2}$ = 0.07, $R_{c}^{2}$ = 0.71	$R_{m}^{2}$ = 0.06, $R_{c}^{2}$ = 0.75	$R_{m}^{2}$ = 0.31, $R_{c}^{2}$ = 0.9	$R_{m}^{2}$ = 0.08, $R_{c}^{2}$ = 0.65	$R_{m}^{2}$ = 0.31, $R_{c}^{2}$ = 0.84
	(Intercept)	**4.08 ± 0.12**	**4.67 ± 0.16**	9.77 ± 5.45	**0.56 ± 0.26**	**−0.77 ± 0.03**	**5.21 ± 1.54**	**6.24 ± 0.1**	**2.75 ± 0.28**	**0.94 ± 0.19**	**0.53 ± 0.06**
Main effect	LAI	—	**0.14 ± 0.03**	**3.88 ± 0.95**	—	—	—	**−0.05 ± 0.01**	**0.03 ± 0.01**	**0.12 ± 0.02**	—
	Soil CN	—	**0.09 ± 0.04**	—	**0.15 ± 0.06**	—	—	—	**0.07 ± 0.03**	—	**0.04 ± 0.01**
	Soil Mg	**−0.1 ± 0.04**	—	**3.49 ± 1.32**	—	—	—	—	—	—	**0.02 ± 0.01**
	Soil N	**0.14 ± 0.05**	**0.1 ± 0.05**	—	—	—	—	—	**0.06 ± 0.03**	—	—
	Soil moist.	—	—	1.95 ± 1.65	—	—	—	**0.03 ± 0.02**	—	—	**0.03 ± 0.01**
	Soil pH	—	—	—	—	—	—	—	**0.05 ± 0.02**	—	—
	Soil temp.	**−0.52 ± 0.08**	**−0.22 ± 0.09**	**7.72 ± 2.61**	—	—	—	**−0.11 ± 0.03**	—	**0.23 ± 0.08**	—
	Temp.	—	—	**5.54 ± 2.42**	**−0.34 ± 0.11**	—	—	—	—	—	—
	Throughfall	**0.07 ± 0.03**	**0.13 ± 0.04**	2.17 ± 1.68	—	—	0.32 ± 0.27	—	—	—	**0.02 ± 0.01**
	GF_pleurocarpous	**−0.36 ± 0.15**	**0.47 ± 0.17**	**12.46 ± 4.51**	—	—	−1.08 ± 2.19	—	0.6 ± 0.39	—	−0.15 ± 0.09
Interaction	LAI:GF	—	**−0.18 ± 0.03**	**−2.72 ± 0.97**	—	—	—	—	—	—	—
	Soil CN:GF	—	—	—	—	—	—	—	**−0.07 ± 0.04**	—	**−0.04 ± 0.02**
	Soil Mg:GF	**0.16 ± 0.06**	—	**−4.3 ± 1.75**	—	—	—	—	—	—	—
	Soil N:GF	**−0.21 ± 0.07**	—	—	—	—	—	—	—	—	—
	Soil moist.:GF	—	—	**−4.25 ± 2.06**	—	—	—	—	—	—	—
	Soil pH:GF	—	—	—	—	—	—	—	**−0.09 ± 0.03**	—	—
	Soil temp.:GF	**0.39 ± 0.1**	—	—	—	—	—	—	—	—	—
	Throughfall:GF	—	—	**−4.22 ± 2.12**	—	—	**−0.77 ± 0.36**	—	—	—	—

Abbreviations: GF, growth form (acrocarpous and pleurocarpous); LAI, leaf area index; Soil moist., soil moisture; Temp., temperature.
